# Cuentas de salud del pasado al presente para una aritmética política^*^

**DOI:** 10.26633/RPSP.2021.72

**Published:** 2021-06-11

**Authors:** Magdalena Rathe, Patricia Hernández, Cornelis Van Mosseveld, Claudia Pescetto, Nathalie Van de Maele

**Affiliations:** 1 Fundación Plenitud Santo Domingo República Dominicana Fundación Plenitud, Santo Domingo, República Dominicana.; 2 Nederlands Interdisciplinair Demografisch Instituut La Haya Países Bajos Nederlands Interdisciplinair Demografisch Instituut, La Haya, Países Bajos.; 3 Consultor La Haya Países Bajos Consultor, La Haya, Países Bajos.; 4 Organización Panamericana de la Salud Washington, DC Estados Unidos de América Organización Panamericana de la Salud, Washington, DC, Estados Unidos de América.; 5 Organización Mundial de la Salud Ginebra Suiza Organización Mundial de la Salud, Ginebra, Suiza.

**Keywords:** Gastos en salud, normas, economía de la salud, historia, planificación en salud, economía, estrategias mundiales, Health expenditures, standards, health economics, history, health planning, economics, world strategies, Gastos em saúde, normas, economia da saúde, história, planejamento em saúde, economia, estratégias mundiais

## Abstract

Este informe describe el proceso de ampliación progresiva de las cuentas de salud para medir los gastos nacionales en salud, desde los primeros intentos de la Asociación Médica Estadounidense en 1926 hasta la actualidad. Se mencionan los hitos en la creación del Sistema de Cuentas de Salud, desde los antecedentes económicos y las acciones iniciales de unos cuantos países y organizaciones a la necesidad de un conjunto de normas de contabilidad para los sistemas de atención de salud y, por último, la consolidación con el Sistema de Cuentas de Salud del 2011. Varias organizaciones internacionales, como la Organización Mundial de la Salud, la Organización para la Cooperación y el Desarrollo Económicos, Eurostat, el Banco Mundial y la Agencia de los Estados Unidos para el Desarrollo Internacional, han sido fundamentales para ampliar los ejercicios nacionales de cuentas de salud y asegurar que estén normalizados, sean comparables y se institucionalicen. Las acciones nacionales para realizar un seguimiento de los gastos en salud no solo han enriquecido los resultados colectivos, sino que se han convertido en un componente importante del liderazgo mundial, al fundamentar las políticas en todo el mundo. Más de 100 países han creado cuentas de salud de conformidad con la norma mundial, y han logrado una mejor comprensión del gasto en salud y de los flujos financieros. Estos resultados son clave para vigilar los avances relativos a las iniciativas nacionales y mundiales, como los Objetivos de Desarrollo Sostenible y la cobertura universal de salud. Todavía quedan retos por delante, como la institucionalización y la calidad de los resultados. También se necesita responsabilidad social para mejorar las fuentes de datos, y aumentar la producción y eluso de las cuentas de salud.

## Abstract (en)

This report traces the progressive expansion of health accounts (HA) to measure national health expenditures, from the first attempts in 1926 by the American Medical Association to the present day. Milestones in the development of A System of Health Accounts (SHA) are covered, from the economic background to initial efforts by a few countries and organizations, to the need for a set of accounting standards for health care systems, and finally, to consolidation with SHA 2011. International organizations, such as the World Health Organization, Organization for Economic Cooperation and Development, Eurostat, the World Bank, and the United States Agency for International Development have been critical to expanding national HA exercises and ensuring that these are standardized, comparable, and become institutionalized. National efforts to track health expenditures have not only enriched collective results, but have become an important component of global leadership, informing policy the world over. More than 100 countries have created HA under the global standard and have gained a better understanding of health spending and financial flows. These results are key for monitoring progress toward national and global initiatives, such as the Sustainable Development Goals and Universal Health Coverage. Challenges remain to be tackled, among them institutionalization and quality of results. Social responsibility for improving data sources and expanding the generation and usability of health accounts are also needed.

## RESUMO

Este informe traça a progressiva expansão das contas da saúde para mensurar os gastos nacionais em saúde, das primeiras tentativas em 1926 pela Associação Americana de Medicina aos dias de hoje. Os marcos do desenvolvimento de um sistema de contas da saúde (SHA) são apresentados: dos antecedentes econômicos, esforços iniciais por parte de alguns países e organizações e necessidade de estabelecer um conjunto de normas contábeis para os sistemas de saúde à consolidação do sistema de contas da saúde (SHA) 2011. As organizações internacionais, como Organização Mundial da Saúde, Organização para a Cooperação e Desenvolvimento Econômico, Eurostat, Banco Mundial e Agência dos Estados Unidos para o Desenvolvimento Internacional, têm sido peças fundamentais para expandir os exercícios de contas nacionais da saúde e assegurar sua padronização, equivalência e institucionalização. Os esforços nacionais para monitorar os gastos em saúde não apenas melhoram os resultados coletivos, mas também são um importante componente de liderança global, servindo de base para políticas no mundo todo. Mais de 100 países criaram contas da saúde segundo o padrão global e têm agora um melhor entendimento do gasto em saúde e fluxos financeiros. Esses resultados são essenciais para monitorar o progresso rumo às iniciativas nacionais e globais, como os Objetivos de Desenvolvimento Sustentável e a cobertura universal de saúde. Existem ainda desafios a serem vencidos, como institucionalização e qualidade dos resultados. Também é preciso responsabilidade social para melhorar as fontes de dados e expandir a geração e a usabilidade das contas da saúde.

Hay una fuerte conexión entre la salud y el desarrollo económico. La inversión en la salud acelera el crecimiento económico y contribuye a reducir la pobreza (1). El gasto en salud puede respaldar el logro de la cobertura universal de salud, y el seguimiento financiero puede contribuir a la eficacia, la eficiencia, la equidad y el empoderamiento de todos los interesados directos (2). El objetivo del seguimiento financiero en el ámbito de la salud es mejorar las estrategias para captar y asignar recursos, reducir el desperdicio de recursos y garantizar la protección financiera. Las cuentas de salud son una herramienta estratégica para llevar a cabo el seguimiento y evaluar las reformas de los sistemas de salud, así como para respaldar la gestión diaria de los recursos. La Organización Mundial de la Salud (OMS) declaró que las cuentas de salud son “información esencial” para el análisis del funcionamiento del sistema de salud, de la misma forma que los datos de mortalidad son esenciales para analizar la situación de salud de un país. En el 2011, la Asamblea Mundial de la Salud aprobó la recomendación de que se presentaran los gastos en salud usando marcos de contabilidad normalizados (3).

Más recientemente, en la *Estrategia para el acceso universal a la salud y la cobertura universal de salud* de la Organización Panamericana de la Salud se hizo referencia concretamente a la necesidad de “aumentar y optimizar el financiamiento” así como“la inversión en salud”, especialmente a partir de fuentes de financiamiento públicas (4). Esta afirmación estimuló el interés por la medición del gasto en salud. Las cuentas de salud necesitan un apoyo político e institucional para ser sostenibles, continuas y técnicamente sólidas. Sin embargo, ¿qué factores determinan si las cuentas de salud se consideran o no esenciales para los sistemas de salud? Tras el reconocimiento de la importancia de las cuentas de salud, ¿se produjo algún cambio en su elaboración o en su uso? ¿Cuál ha sido la evolución de las cuentas de salud en el mundo?

El objetivo de este artículo es presentar los hitos alcanzados durante el desarrollo de un Sistema de Cuentas de Salud, desde su inicio hasta la actualidad. Los períodos de desarrollo se describen de la siguiente forma: *a*) las bases, es decir, el fundamento económico común de las cuentas nacionales; *b*) las acciones iniciales realizadas por unos pocos países y organizaciones; *c*) la propuesta del primer Sistema de Cuentas de Salud como norma para la contabilidad del sistema de salud, y *d*) la consolidación con el Sistema de Cuentas de Salud del 2011 (conocido como el SHA 2011).

RECUADRO 1. **Aritmética política: el estudio de las estadísticas económicas y demográficas de un estado**

Petty explicó que esta forma de ciencia usaría tan solo fenómenos cuantificables y buscaría la precisión cuantitativa, en lugar de depender de comparativos o superlativos. Como economista moderno, demostró sus afirmaciones mediante datos y estadísticas, en lugar de datos no comprobados (5).

**FIGURA 1.**
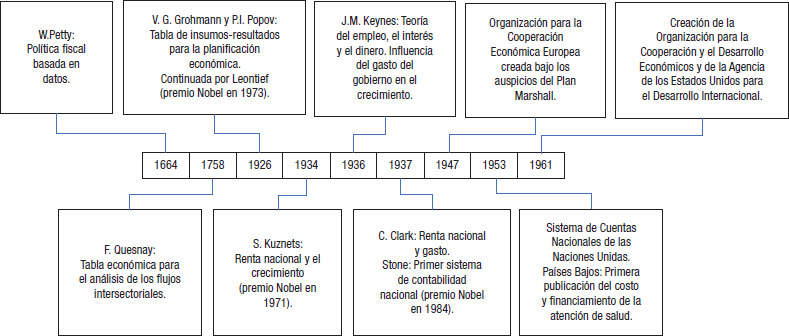
Bases del Sistema de Cuentas de Salud

***Fuente:*** Elaborado por los autores a partir de datos del estudio.

## BASES

En todas las grandes culturas de la civilización hay registros contables. El origen de las cuentas macroeconómicas y sociales puede vincularse a la filosofía aristotélica, que estableció el valor como la unidad básica para el intercambio y la relación entre el precio y la cantidad para medirlo. Cerca de veinte siglos después, en 1664, William Petty redescubrió la idea e inició la historia de las cuentas macroeconómicas (recuadro 1).

El trabajo de autores como Quesnay, Marx, Popov, Kuznets, Keynes, Clark y Stone (5) contribuyó a desarrollar lo que ahora se conoce como un “Sistema de Cuentas Nacionales” (SCN). En el caso de las instituciones, esto se vinculó con la Sociedad de Naciones, que posteriormente se convirtió en las Naciones Unidas, y la Organización Europea para la Cooperación Económica (OECE), que luego pasó a ser la Organización para la Cooperación y el Desarrollo Económicos (OCDE). Como se muestra en la figura 1, la estructura conceptual del SCN se modificó en 1960, 1964, 1968, 1993 y 2008 (6). El equivalente a nivel de la Unión Europea (UE), el Sistema Europeo de Cuentas (SEC), se modificó en 1970, 1995 y 2010, y es obligatorio para los Estados Miembros de la UE (7). El SCN de 1993 incluyó un capítulo sobre cuentas satélites, que reproducía o adaptaba el sistema central para dar respuesta a cuestiones sobre políticas en apartados específicos; posteriormente, el SCN del 2008 (8) se refirió al Sistema de Cuentas de Salud como la cuenta satélite para la salud.

## ACCIONES INICIALES

### Pioneros

Estados Unidos y varios países de la Unión Europea han sido pioneros en la elaboración y el uso de cuentas de salud para la toma de decisiones sobre políticas.

En 1932, un grupo de trabajo de la Asociación Médica Estadounidense publicó un informe sobre el gasto en salud correspondiente al año 1926 (9). Los programas federales de salud de Estados Unidos y su Administración de Seguridad Social aplicaron la contabilidad a las finanzas de la salud alrededor de los años sesenta. Desde entonces, la Administración Financiera de Asistencia Sanitaria (ahora Centro de Servicios de Medicare y Medicaid) ha publicado anualmente los cálculos de los flujos de financiamiento sanitario tanto públicos como privados del país con el mayor gasto en salud del mundo. Estos cálculos incluyen cuánto dinero se asigna a la salud, quién lo paga y a qué servicios se destina. Los cálculos existentes hasta la fecha abarcan también el gasto directo de los hogares, los ingresos de los hospitales y los grupos beneficiarios. Los informes contribuyen al análisis de los problemas principales, como el aumento del gasto en salud, las importaciones y exportaciones de servicios y medicamentos, el gasto en recursos humanos y el análisis de las crisis económicas (10).

En Francia, el consumo de atención de salud se ha medido desde 1950 (11). El Instituto Nacional de Estadística y el Ministerio de Salud han publicado los datos desde 1979 en adelante. Las cuentas se han usado en el debate sobre políticas públicas para abordar en mayor profundidad aspectos como el consumo de medicamentos y el costo de la enfermedad.

La Oficina de Estadística de los Países Bajos publicó el primer informe de ese país sobre los costos y el financiamiento de la salud en 1957 (con datos correspondientes a 1953), con estimaciones de los proveedores y del financiamiento. Desde 1972, se ha publicado anualmente el gasto en salud y, hasta el año 2000, estos informes incluían la estructura de costos de los proveedores y las inversiones. A partir del 2000, los informes comprenden los datos del gasto en salud y el gasto social que abordan las necesidades de políticas del Ministerio de Salud y respaldan las acciones destinadas a aproximarse a las Cuentas Nacionales (CN). El Ministerio se encarga de la atención de salud y la asistencia social, y ha elaborado un análisis político de las cuentas desde comienzos de la década de los ochenta (12).

La Oficina de Estadística Alemana publicó el gasto en salud correspondiente a la década de los ochenta, con un intervalo de tiempo transcurrido de cinco años respecto al año de los datos. En la década de los noventa, los datos se presentaron a los encargados de adoptar las decisiones cinco meses después del cierre contable de cada año. Estos informes incluían inicialmente datos de financiamiento de la salud, información sobre quién prestaba los servicios y con qué insumos y, posteriormente, un análisis de los recursos humanos y de las enfermedades (13).

### Organizaciones internacionales

En los años sesenta, la OMS patrocinó un grupo de estudio de seis países (14) con el objetivo de evaluar sus medios económicos y sus proveedores de servicios de salud (quién presta qué servicios y con qué insumos). A este estudio le siguió otro de 14 países en 1967 (15), con una perspectiva de sistemas. A lo largo de las décadas siguientes, las mediciones se centraron en las acciones nacionales destinadas a financiar entidades de atención de salud públicas y privadas. Sin embargo, la falta de un marco de referencia general homogéneo y la necesidad de una mayor comparabilidad eran evidentes.

En 1977, la OCDE publicó la primera comparación internacional de Poullier basada en los conceptos de cuentas nacionales (16), en la que se presentaba una década de datos de gastos en salud y posibles escenarios futuros. Su segundo compendio (17) incluyó datos de gastos, precios y volumen, y promovió la idea de un seguimiento anual de series históricas de indicadores de financiamiento, producción y resultados, para respaldar las políticas de salud. Desde 1991, este esfuerzo se ha consolidado con el informe anual de datos de salud disponible en línea en http://www.oecd.stat/ (para los Estados Miembros y países asociados).

Desde los años ochenta, la OCDE ha organizado reuniones anuales sobre datos y análisis metodológicos, incluidas las cuentas de salud. Los países se han hecho cargo progresivamente de estas acciones de recopilación de datos sobre el gasto en atención de salud.

En 1993, el Banco Mundial publicó un informe sobre la importancia del gasto en salud (18) que impulsó el apoyo a las cuentas de salud y alentó a algunos países a que siguieran el ejemplo.

### Avances en el resto del mundo

Dos elementos promovieron el desarrollo de las cuentas de salud en el mundo: la necesidad de un análisis económico de la salud vinculado a las reformas del sector de la salud y el apoyo de donantes dispuestos a facilitar el proceso. En América Latina, el primer país que elaboró cuentas de salud fue México en 1994. Las cuentas de salud formaron parte de un análisis orientado a sostener la reforma del sector de la salud sustentado en la evidencia (19). Este ejercicio determinó quién paga la atención de salud, de dónde proceden los fondos y quién presta qué servicios y con qué insumos. En Perú, las primeras cuentas de salud comenzaron en 1996 y fueron fundamentales para sustentar las reformas del sector de la salud en ese país (20).

En 1996, la Organización Panamericana de la Salud (OPS) y el proyecto Partners for Health Reform, con el financiamiento de la Agencia de los Estados Unidos para el Desarrollo Internacional (USAID), prestaron apoyo a los siguientes ocho países de América Latina y el Caribe) en la elaboración conjunta de cuentas de salud: Bolivia, Ecuador, El Salvador, Guatemala, Honduras, México, Nicaragua y República Dominicana. La idea era que América Latina aprovechara la experiencia de Estados Unidos, en donde los sistemas de salud están más fragmentados y hay una parte más importante del gasto que es de carácter privado (21). De forma asociada, la OPS, el Banco Mundial, el Banco Interamericano de Desarrollo (BID) y la USAID propusieron que hubiera cuentas de salud que abarcaran toda América Latina en un plazo de cinco años. Tras dos años, la experiencia de América Latina y el Caribe puso de manifiesto la necesidad de una mayor comparabilidad de los resultados de diferentes países.

**FIGURA 2.**
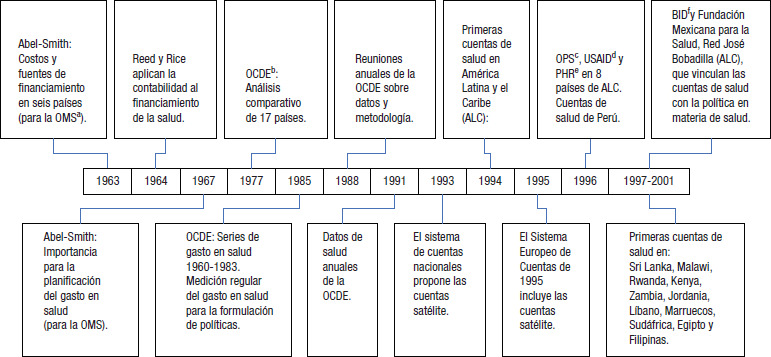
Acciones iniciales de los diversos países y organizaciones con respecto al empleo de las cuentas de salud para medir el gasto en salud, 1963–2001

^a^Organización Mundial de la Salud.

^b^Organización para la Cooperación y el Desarrollo Económicos.

^c^Organización Panamericana de la Salud.

^d^Agencia de los Estados Unidos para el Desarrollo Internacional.

^e^Partners for Health Reform.

^f^Banco Interamericano de Desarrollo.

***Fuente:*** Elaborado por los autores a partir de datos del estudio.

En el resto del mundo, el impulso de la USAID y la OMS para guiar la elaboración de cuentas de salud ayudó a crear redes regionales en África, Asia y Oriente Medio. Algunas de estas redes están institucionalizadas hoy en día (figura 2).

## PRIMERA NORMA

La preocupación por la comparabilidad entre los Estados Miembros de la OCDE condujo al proyecto de “paquetes comparables comunes”, en el que se evaluaron conjuntos de actividades que podían usarse como elementos básicos comunes. Este proyecto formó parte del trabajo preliminar para el Sistema de Cuentas de Salud (22). En respuesta a la necesidad de una mayor uniformidad metodológica, la OCDE publicó la versión 1.0 (23) en el año 2000. El marco triaxial se centró en el consumo final y tuvo como objetivo contribuir a la toma de decisiones del sistema de salud mediante el análisis de los gastos según los servicios (funciones), su financiamiento y sus proveedores. Estas clasificaciones se basaron en las cuentas nacionales (clasificaciones según el sector y la finalidad), e incluyeron datos consolidados de los gastos de capital. En el manual se proponía información adicional sobre el comercio y la distribución del gasto según la edad, el sexo y la enfermedad, pero esto no se agregó a la estructura de la clasificación. El Sistema de Cuentas de Salud facilitó la comparabilidad internacional.

En la mayoría de los países de la OCDE, el trabajo en las cuentas de salud se vinculó a los organismos de estadística. En los países de ingresos bajos y medianos las acciones fueron promovidas por los Ministerios de Salud. En el 2003, la OMS, el Banco Mundial y la USAID publicaron la *Guía del productor de cuentas nacionales de salud* para explicar la manera de poner en práctica el Sistema de Cuentas de Salud (24). Esta guía también incluía costos de los recursos (insumos) y fuentes de financiamiento, y resaltaba la distribución según el grupo beneficiario: posición socioeconómica, ubicación, enfermedad, edad y sexo.

El Banco Mundial y otros bancos de desarrollo regionales han respaldado las cuentas de salud, incluyéndolas incluso como requisito en algunos préstamos. Esta práctica continúa hoy en día en instituciones como el Fondo Mundial. Las oficinas regionales de la OMS, la USAID y los propios países desplegaron iniciativas a fin de poner en marcha las estimaciones de una manera normalizada y comparativa.

El *Informe sobre la salud en el mundo* correspondiente al año 2000 (25), de la OMS, inició la publicación anual de indicadores del gasto en salud de todos los Estados Miembros (26). Actualmente, los datos se publican en la base de datos mundial sobre gasto sanitario, la primera iniciativa pública mundial de este tipo. Sus datos se reproducen en publicaciones asociadas del Banco Mundial y otros organismos.

### Labor de los países por región

Los países han impulsado conjuntamente el proceso del Sistema de Cuentas de Salud de distintas maneras. La Red de Cuentas de Salud de Asia-Pacífico (27) se puso en marcha a comienzos de la década del 2000 y promovió las cuentas de salud de calidad a través de acciones metodológicas, de colaboración y de difusión, para facilitar la toma de decisiones y la apropiación por parte de los países. Esta red colabora actualmente con el Centro de Políticas de Corea (OCDE) y con las oficinas regionales de Asia Sudoriental y el Pacífico Occidental de la OMS. La OCDE, la OMS y los Estados Miembros de la Unión Europea han utilizado el cuestionario conjunto de cuentas de salud del Sistema de Cuentas de Salud desde el 2005 para una notificación normalizada, eficiente y colaborativa.

En el 2008 se creó la Red de las Américas de Cuentas de Salud (REDACS) (28). Esta red cuenta con el apoyo de la OMS, el Instituto Tecnológico de la Universidad de Santo Domingo (Santo Domingo, República Dominicana), el Ministerio de Salud de República Dominicana, la Fundación Mexicana para la Salud (Ciudad de México, México), el BID y la USAID, y está coordinada por la Fundación Plenitud, en el marco del Observatorio de Salud de América Latina y el Caribe (Ciudad de México, México). La red tiene como objetivo tener cuentas de salud de alta calidad para la toma de decisiones mediante el Sistema de Cuentas de Salud.

La OPS publicó y promovió el manual sobre cuentas satélite en el 2005 (29) con la finalidad de ampliar el marco central aplicado a los sistemas de salud. Se centra en la producción, el consumo y la generación de ingresos. Algunos países como Brasil, Ecuador y Portugal han elaborado series completas de datos con este modelo. Chile y Paraguay elaboraron series relativas al sector público. Otros países, como Noruega, aplicaron directamente el marco central vinculado al Sistema de Cuentas de Salud. Las cuentas satélite mostraron ser complementarias respecto al Sistema de Cuentas de Salud, al proporcionar puntos de vista diferentes, pero importantes para los encargados de las decisiones, sobre el sistema de salud (30). Las cuentas satélite han respaldado las deliberaciones con los ministerios de economía y finanzas para defender la asignación de recursos públicos.

Desde el año 2000, la Región de África ha estado inmersa en la preparación del Sistema de Cuentas de Salud, con el apoyo de la OMS. En la actualidad, África occidental es la subregión en la que las cuentas de salud han alcanzado la máxima participación real, con vínculos pertinentes con las políticas nacionales y regionales, como la Declaración de Abuya (31). Este acuerdo incluye el objetivo de dedicar anualmente un 15% del gasto gubernamental a la atención de salud.

**FIGURA 3.**
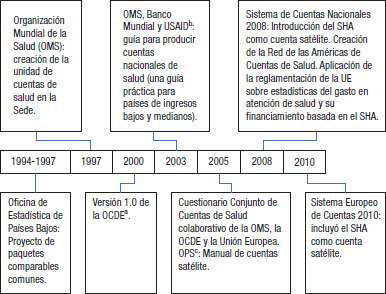
Normalización para lograr un Sistema de Cuentas de Salud, 1994–2010

^a^Organización para la Cooperación y el Desarrollo Económicos.

^b^Agencia de los Estados Unidos para el Desarrollo Internacional.

^c^Organización Panamericana de la Salud.

Se ha realizado una labor considerable para dar seguimiento al uso de los recursos destinados a programas específicos como los de la salud reproductiva (32, 33), la infección por el VIH/sida (34) y la tuberculosis y la malaria (35), lo que se denomina subcuentas, ya que tienen como finalidad el seguimiento de los recursos relativos a una enfermedad específica. Con demasiada frecuencia, el apoyo inicial proporcionado por donantes económicos para combatir enfermedades concretas luego disminuye gradualmente. Esto ha llevado a la atención de los responsables de las políticas la necesidad de un financiamiento sostenible y respaldado por el gobierno (figura 3).

## CONSOLIDACIÓN CON EL SISTEMA DE CUENTAS DE SALUD DEL 2011

En las deliberaciones en las reuniones anuales del grupo de trabajo internacional de Eurostat (Grupo de Trabajo sobre Salud Pública, Comisión Europea, Luxemburgo), la OCDE (Reunión de Expertos en Cuentas de Salud) y la OMS se pusieron de manifiesto las interpretaciones diversas y divergentes del marco de referencia de la versión 1.0. La experiencia obtenida en la labor con la versión 1.0 permitió a los grupos establecer la necesidad de una revisión. Eurostat, la OCDE y la OMS coordinaron el proceso de revisión mediante un equipo internacional de expertos en cuentas de salud y designaron a la OCDE para que actúe como secretaría. Las consultas incluyeron reuniones regionales y mundiales con 130 países participantes y con organismos internacionales, como el Banco Mundial y el BID. El punto de vista de los expertos se complementó con algunas experiencias piloto (36) para evaluar la viabilidad y asegurar una migración adecuada de las series de datos de gasto existentes a la nueva propuesta. El proceso de revisión empezó en el 2006 y duró cinco años, hasta producir el Sistema de Cuentas de Salud del 2011 (conocido como SHA 2011 por su sigla en inglés) como la nueva norma mundial avalada por la Oficina Estadística de Naciones Unidas. De manera complementaria y simultánea, el Banco Mundial publicó una guía para la institucionalización de las cuentas de salud (37).

***Fuente:*** Elaborado por los autores a partir de datos de estudios.

El objetivo del SHA 2011 es lograr una mayor comparabilidad y evaluar el funcionamiento, con independencia de la diversidad de sistemas de salud existentes en el mundo.

Su propósito es aumentar la normalización, la uniformidad y la pertinencia para las políticas, así como facilitar la generación continua de cuentas de salud y su uso en la toma de decisiones. Las diferencias más importantes del SHA 2011 respecto a la versión 1.0 son una vinculación más fuerte con las funciones de financiamiento (recaudación de ingresos, mancomunación y asignación/compra de servicios) con clasificaciones contables que representen de manera más sólida el sistema de financiamiento de la salud, una clasificación de la formación de capitales mixtos para analizar mejor el gasto actual y de capital, y algunas mejoras menores relativas a la prevención, las organizaciones sin fines de lucro y los recursos externos. En aras de una mayor uniformidad, el SHA 2011 propone una distribución completa del gasto por grupos principales de enfermedad (establecidos según la décima edición de la Clasificación Internacional de Enfermedades o la carga mundial de enfermedad) en lugar de por subcuentas.

**FIGURA 4.**
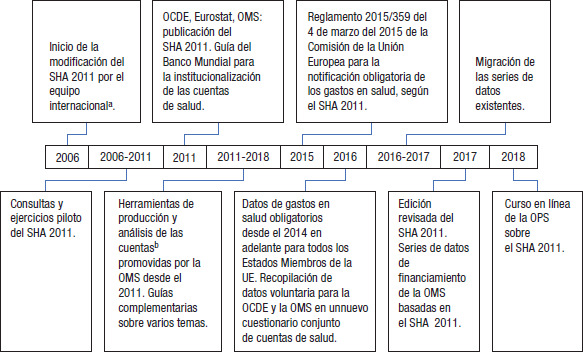
Consolidación del Sistema de Cuentas de Salud del 2011 (SHA 2011), 2006–2018

^a^ Equipo Internacional de Expertos en Cuentas de Salud integrado por la Organización para la Cooperación y el Desarrollo Económicos (OCDE), la Organización Mundial de la Salud (OMS) y Eurostat, en el que la OCDE actúa como secretaría.

^b^ Herramienta de producción de cuentas de salud (“Health Accounts Production Tool”) y herramienta de análisis de cuentas de salud (“Health Accounts Analysis Tool”).

***Fuente:*** Elaborado por los autores a partir de datos del estudio.

En su reglamentación del 2015 sobre los gastos de atención de salud (38), la Unión Europea indicó que a partir del 2016 debía usarse el SHA 2011 para clasificar las funciones/servicios, los proveedores de servicios de salud y el financiamiento de la atención de salud. La OCDE, Eurostat y la OMS han elaborado guías complementarias y herramientas sobre temas de interés, como el financiamiento, la prevención, la administración, los medicamentos, el capital y las enfermedades. La OMS creó dos herramientas informáticas para facilitar la generación de las cuentas, una herramienta de producción de cuentas de salud y una herramienta de análisis de las cuentas de salud, para respaldar la institucionalización, producción y análisis de los datos, y para simplificar el uso de resultados. En el 2017 se presentó una versión modificada del SHA 2011 (39) que mantenía el contenido, pero lo exponía con mayor claridad (figura 4).

### Retos de la institucionalización

Los equipos de cuentas de salud se centran en la institucionalización del SHA 2011, su método, principios, datos y registros. Las series existentes se han hecho compatibles con el SHA 2011, al tiempo que se incorporaron los cambios propuestos por la nueva norma. Todo ello ha llevado a una mejora de la calidad de las estimaciones. La OMS, la UE y la OCDE comenzaron la recopilación de datos según el SHA 2011 en el año 2016, y la presentación de información en el 2017. La participación es obligatoria para los Estados Miembros de la UE.

Hasta la fecha, muchos países han generado cuentas, pero no han logrado institucionalizarlas. La institucionalización no es tan solo para la recopilación y producción regular de los datos, sino también para el uso de los resultados en la toma de decisiones de políticas. En 1980, cerca de 15 países, principalmente de la OCDE, habían producido cuentas de salud; al llegar al 2000, el número había aumentado a 87; en el 2010, a 130 países; y en el 2017, a cerca de 160 países (40). En el 2017, un total de 72 países no pertenecientes a la OCDE presentaron como mínimo un ejercicio del SHA 2011 y de ellos, de 30 a 40 países producen cuentas de salud de forma periódica e institucionalizada. Sin embargo, hay todavía algunos países que lo han hecho una sola vez, que no generan cuentas de forma continuada o que no usan las cuentas de salud para la formulación de políticas. Esto implica que todavía se necesita una mayor institucionalización.

## DISCUSIÓN Y CONCLUSIONES

La norma mundial actual, el SHA 2011, es el resultado de un largo proceso de elaboración que se inició con los primeros intentos de contabilización del gasto en salud a mediados del siglo XX, sin ninguna clasificación específica, y condujo al manual publicado en París en el 2011. El SHA 2011 es compatible con las cuentas nacionales y fue elaborado por expertos en financiamiento de la salud, sistemas de salud y cuentas de salud, que colaboraron para alcanzar un consenso respecto a las clasificaciones y definiciones.

Con el SHA 2011 es viable generar indicadores que vinculen los recursos con los resultados de salud u otras variables de interés para la formulación de políticas.

Algunas cuestiones clave como la equidad, la eficiencia y la calidad pueden evaluarse de manera periódica e institucionalizarse para aumentar la transparencia y la rendición de cuentas, y fortalecer la gobernanza del sistema de salud. El SHA 2011 sirve de puente para las políticas de financiamiento de la salud y su análisis.

La Comisión sobre la Información y la Rendición de Cuentas para la Salud de la Mujer y el Niño (41) ha propuesto el uso de las cuentas de salud para el seguimiento de la supervisión de los gastos en salud reproductiva, neonatal, materna e infantil (42). Otras iniciativas conexas, como la Cuenta Regresiva para el 2015 (seguimiento de los progresos en relación con la supervivencia materna, neonatal e infantil, conocida actualmente como la Cuenta Regresiva para el 2030), incluyen datos de seguimiento del financiamiento y los recursos (43). Esta iniciativa y otras similares han subrayado la importancia de las decisiones que están respaldadas por intervenciones basadas en la evidencia.

En un contexto más amplio, el avance hacia la atención universal de salud requiere información económica esencial, y el grado de protección económica proporcionada a los hogares es un aspecto crucial. Se reconoce que el nivel del gasto pagado de manera directa por los propios usuarios (o gasto de bolsillo) constituye un reto importante para el acceso a la atención de salud. Aunque no se aborda explícitamente en los Objetivos de Desarrollo del Milenio, la protección económica de los hogares constituye una meta de los Objetivos de Desarrollo Sostenible. En la Región de las Américas, el nivel del gasto de bolsillo ha sido considerado en la *Estrategia para el acceso universal a la salud y la cobertura universal de salud* (4) como una barrera para el acceso, y representa la fuente de financiamiento más ineficiente.

Las cuentas de salud proporcionan una información esencial, por medio de un sistema homogéneo y normalizado a nivel internacional. Las cuentas de salud aportan datos comparables a lo largo del tiempo y entre distintos países, con independencia de las diferencias existentes en la organización de los sistemas de salud. La mejora de la medición ha pasado a ser de capital importancia, por lo que se deben aplicar algunas medidas clave, como aumentar la calidad de la información que se presenta, la capacitación y las estrategias de control (44). Se espera que la convergencia de los esfuerzos realizados en relación con el plan de acción mundial de las Naciones Unidas para los datos sobre desarrollo sostenible (45) produzca cifras de mayor calidad y una mejora de los registros y, por consiguiente, mejores fuentes de información.

En el SHA 2011 se alienta a los países a que desarrollen un seguimiento estandarizado de los gastos en salud, como una función fundamental de rectoría de las autoridades nacionales de salud. De hecho, la función de la OCDE y la OMS, así como el apoyo continuo de asociados estratégicos, como la USAID, el Banco Mundial y el BID, son cruciales para alcanzar datos estadísticos comparables. Para mejorar los beneficios del marco y las directrices disponibles, es deseable que haya traducciones oficiales a los principales idiomas.

El uso de los datos de las cuentas de salud no siempre ha sido reconocido y citado en las referencias. Sin embargo, las cuentas de salud proporcionan un panorama integral de todos los flujos financieros de todas las partes involucradas. Esto difícilmente se logra con otros enfoques, como los exámenes del gasto público, los presupuestos estatales o del Ministerio de Salud y las cuentas nacionales. El potencial de las cuentas de salud continúa mejorando con la experiencia, como ha ocurrido en el caso de las iniciativas de la OCDE y la OMS destinadas a concretar y estandarizar las cuentas relativas a la atención primaria.

La Región de las Américas afronta problemas que se registran también en otras zonas, como la escasa normalización de los registros primarios del gasto y, una vez elaboradas las cuentas, una falta de memoria institucional para acelerar la continuidad y la permanencia. La tarea de institucionalizar las cuentas (producción continua y uso estratégico de los resultados) requiere un compromiso nacional, una iniciativa hábil de desarrollo y un deseo de transparencia que vaya más allá de la esfera pública e incluya también a las partes involucradas del ámbito privado. Las cuentas de salud de buena calidad son el resultado del compromiso de un país en todas las esferas sociales y políticas. América Latina y el Caribe tienen la oportunidad de mejorar el uso complementario de cuentas satélite del SCN así como el empleo del SHA 2011 para la formulación de políticas.

En general, las entidades y las personas que elaboran las cuentas de salud no son los responsables de las políticas ni los demás usuarios. Las cuentas de salud son necesarias para mejorar el conocimiento y promover la comunicación entre estas diversas partes involucradas. El fomento del uso de cuentas para apoyar las políticas, por parte de los responsables de las políticas en cada país, así como de los organismos internacionales, es fundamental para lograr su institucionalización. En la Región de las Américas, la OPS/OMS continúa teniendo un papel importante que desempeñar en este sentido.

## Declaración.

Los autores son los únicos responsables de las opiniones expresadas en el manuscrito, que pueden no reflejar necesariamente la opinión o la política de *RPSP/PAJPH* o de la OPS.
